# Radiomics analysis of contrast-enhanced CT for staging liver fibrosis: an update for image biomarker

**DOI:** 10.1007/s12072-022-10326-7

**Published:** 2022-03-28

**Authors:** Jincheng Wang, Shengnan Tang, Yingfan Mao, Jin Wu, Shanshan Xu, Qi Yue, Jun Chen, Jian He, Yin Yin

**Affiliations:** 1 Department of Hepatobiliary Surgery, Nanjing Drum Tower Hospital, The Affiliated Hospital of Nanjing University Medical School, 321 Zhongshan Road, Nanjing, 210008 Jiangsu Province China; 2 Department of Hepatobiliary Surgery, Nanjing Drum Tower Hospital Clinical College of Nanjing Medical University, Nanjing, China; 3 Department of Nuclear Medicine, Nanjing Drum Tower Hospital, The Affiliated Hospital of Nanjing University Medical School, 321 Zhongshan Road, Nanjing, 210008 Jiangsu Province China; 4 Department of Pathology, Nanjing Drum Tower Hospital, The Affiliated Hospital of Nanjing University Medical School, Nanjing, China; 5 Preparatory School for Chinese Students To Japan, The Training Center of Ministry of Education for Studying Overseas, Changchun, China

**Keywords:** Radiomics, Contrast-enhanced CT, Liver fibrosis, Prediction model, Cirrhosis, Noninvasive, Machine learning, Obuchowski index, Calibration, Decision curve analysis

## Abstract

**Background:**

To establish and validate a radiomics-based model for staging liver fibrosis at contrast-enhanced CT images.

**Materials and methods:**

This retrospective study developed two radiomics-based models (R-score: radiomics signature; R-fibrosis: integrate radiomic and serum variables) in a training cohort of 332 patients (median age, 59 years; interquartile range, 51–67 years; 256 men) with biopsy-proven liver fibrosis who underwent contrast-enhanced CT between January 2017 and December 2020. Radiomic features were extracted from non-contrast, arterial and portal phase CT images and selected using the least absolute shrinkage and selection operator (LASSO) logistic regression to differentiate stage F3–F4 from stage F0–F2. Optimal cutoffs to diagnose significant fibrosis (stage F2–F4), advanced fibrosis (stage F3–F4) and cirrhosis (stage F4) were determined by receiver operating characteristic curve analysis. Diagnostic performance was evaluated by area under the curve, Obuchowski index, calibrations and decision curve analysis. An internal validation was conducted in 111 randomly assigned patients (median age, 58 years; interquartile range, 49–66 years; 89 men).

**Results:**

In the validation cohort, R-score and R-fibrosis (Obuchowski index, 0.843 and 0.846, respectively) significantly outperformed aspartate transaminase-to-platelet ratio (APRI) (Obuchowski index, 0.651; *p* < .001) and fibrosis-4 index (FIB-4) (Obuchowski index, 0.676; *p* < .001) for staging liver fibrosis. Using the cutoffs, R-fibrosis and R-score had a sensitivity range of 70–87%, specificity range of 71–97%, and accuracy range of 82–86% in diagnosing significant fibrosis, advanced fibrosis and cirrhosis.

**Conclusion:**

Radiomic analysis of contrast-enhanced CT images can reach great diagnostic performance of liver fibrosis.

**Supplementary Information:**

The online version contains supplementary material available at 10.1007/s12072-022-10326-7.

## Background

Liver fibrosis is an important cause of morbidity and mortality in patients with chronic insults (e.g. viral hepatitis, alcohol and non-alcoholic fatty liver diseases [NAFLD]) and complications mainly occur in advanced fibrosis [1]. Fibrosis staging is an essential step in the clinical assessment of patients with chronic liver disease to identify those who require treatment [2]. Liver biopsy is the current reference method for staging fibrosis, but it has defects including invasiveness, sample biases and interobserver variability [3–6]. Therefore, there is a need for noninvasive and accurate methods for staging liver fibrosis.

2018 practice guidance of the American Association for the Study of Liver Diseases (AASLD) recommended multiphase CT or MRI for initial diagnostic testing in at-risk patients with abnormal surveillance test results [7]. Compared to MRI, CT offers unique advantages including low cost, fewer contradictions, nearly ubiquitous availability and whole organ imaging capacity [8]. To date, several studies have evaluated the ability of contrast-enhanced CT imaging to determine the severity of liver fibrosis [8–10]. However, the sample sizes of these studies were not big enough and that may not be sufficient for development and validation of models.

In the era of personalized medicine, radiomics has allowed large number of quantitative features to be extracted from images that provide information on shape, signal intensity and texture [11, 12]. Our previous study established and validated a radiomics-based model at non-contrast CT for the prediction of cirrhosis in patients with hepatitis B virus (HBV) [13]. We hypothesized that a model based on radiomics features extracted from contrast-enhanced CT images may improve the staging of liver fibrosis. Therefore, the aim of this study was to develop and validate a radiomics model for the prediction of liver fibrosis using contrast-enhanced CT in the liver.

## Materials and methods

This retrospective study was approved by the institutional review board of our institution, and the requirement for written informed consent was waived.

### Patients

Among the 1779 consecutive patients who underwent abdominal contrast-enhanced CT at our institution between January 2017 and December 2020, patients over 18 years who had available pathologic records of liver fibrosis within 3 months of liver images at 1.5 mm thickness were retrospectively reviewed. Of 927 eligible patients, 484 were excluded due to conditions that may interfere with the extraction of radiomic features of their nontumorous right hepatic lobes, including large (≥ 10 cm) or multiple (≥ 5) hepatic masses (*n* = 276), a tumor thrombus in the portal vein larger than the segmental branch (*n* = 63), bile duct obstruction (*n* = 29), previous surgical resection on the right hepatic lobe (*n* = 28), poor image quality because of metal or respiratory motion artifacts (*n* = 60) and incomplete clinical data (*n* = 28).

A total of 443 patients, including 345 men (median age, 56 years; age range, 28–86 years) and 98 women (median age, 61 years; age range, 40–84 years), were finally included in this study cohort. This cohort was randomized in a three-to-one ratio into training and validation cohorts, respectively, using computer-generated random numbers without matching of any patient characteristics.

**Fig. 1:**
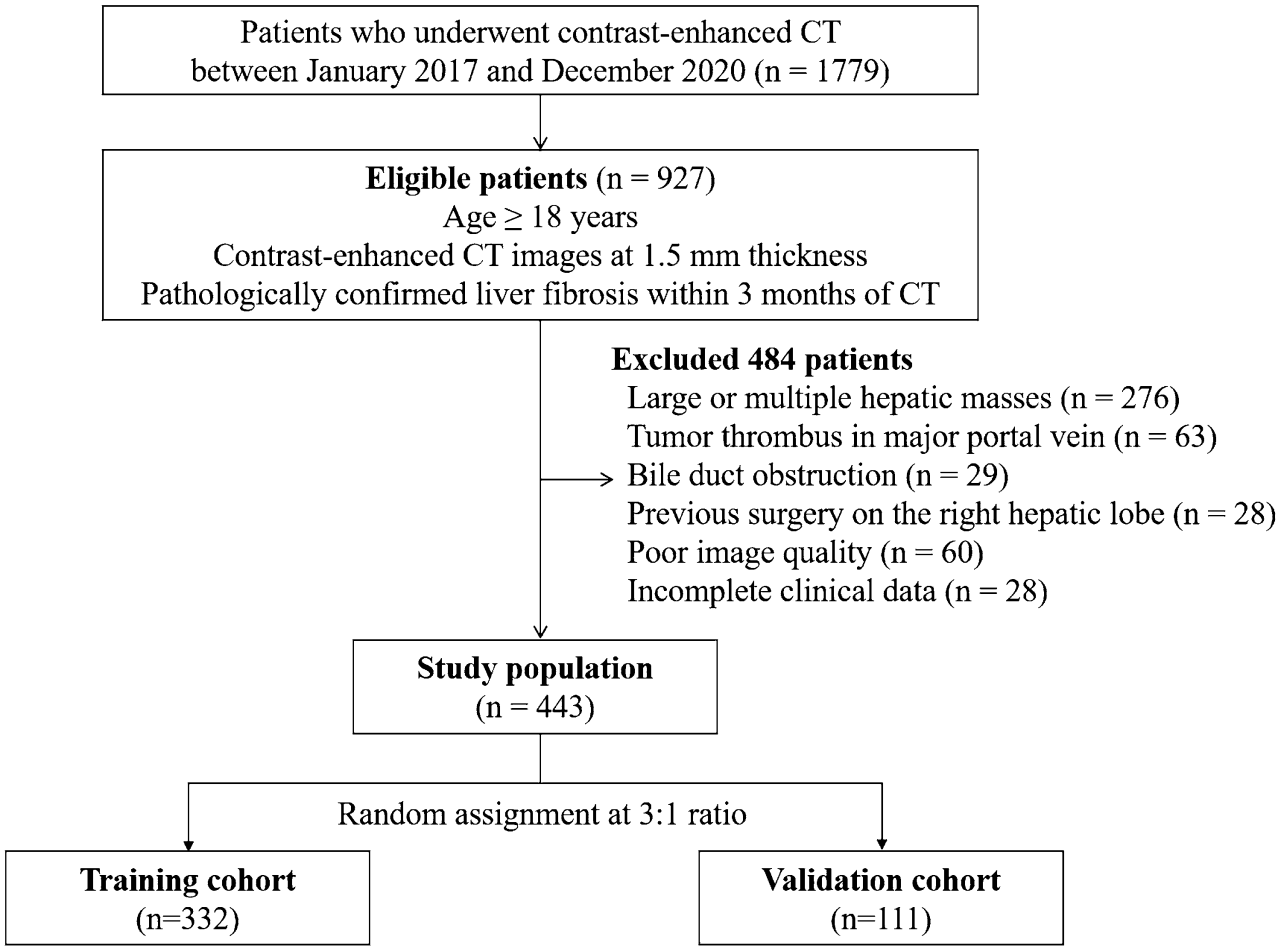
Patient selection flow chart. *CT* computed tomography

332 patients (median age, 59 years; age range, 28–86 years; 256 men [median age, 58 years; age range, 28–86 years] and 76 women [median age, 63 years; age range, 47–84 years]) were included in the training cohort and 111 (median age, 56 years; age range, 35–75 years; 89 men [median age, 56 years; age range, 35–75 years] and 22 women [median age, 55 years; age range, 40–68 years]) were in the validation cohort. The flow diagram for the study population is shown in Fig. 1 and Table 1 shows the demographic and clinical characteristics of the cohorts. The median interval between CT images and pathologic evaluation was 15 days ± 18 (standard deviation; range, 1–76 days).

**Table 1:** Patient characteristics

Parameter	Development (*n* = 332)	Validation (*n* = 111)	*p* value
Sex			0.60
No. of men	256 (77.1)	89 (80.2)	
No. of women	76 (22.9)	22 (19.8)	
Age (years)*	59 (51–67)	58 (49–66)	0.49
Men	57 (50–66)	58 (49–66)	
Women	63 (56–69)	56 (54–59)	
Underlying liver disease			0.19
Hepatitis B	176 (53.0)	70 (63.1)	
Hepatitis C	13 (3.9)	6 (5.4)	
NAFLD	11 (3.3)	2 (1.8)	
Primary biliary cirrhosis	6 (1.8)	0 (0)	
None	126 (38.0)	33 (29.7)	
Hepatic tumor			0.84
HCC	121 (36.5)	41 (36.9)	
Other malignancy	21 (6.3)	5 (4.5)	
Hemangioma	66 (19.9)	20 (18.0)	
None	124 (37.3)	45 (40.6)	
Pathologic confirmation method			0.54
Percutaneous liver biopsy	74 (22.3)	29 (26.1)	
Liver resection	232 (69.9)	76 (68.5)	
Liver transplantation	26 (7.8)	6 (5.4)	
Laboratory findings*			
AST (IU/mL)	29.8 (20.9–39.3)	29.2 (21.6–46.6)	0.26
ALT (IU/mL)	27.3 (19.3–45.2)	32.1 (21.2–49.5)	0.16
Total bilirubin (ng/mL)	12.7 (9.5–17.8)	13.7 (9.7–17.6)	0.42
Platelet count (10^9^/L)	138 (95–184)	143 (87–180)	0.91
INR	1.03 (0.97–1.10)	1.01 (0.96–1.08)	0.31
APRI	0.6 (0.3–1.0)	0.6 (0.3–1.3)	0.30
FIB-4	2.4 (1.6–4.1)	2.7 (1.7–4.4)	0.96
Metavir fibrosis stage			0.10
F0	43 (13.0)	10 (9.0)	
F1	60 (18.1)	14 (12.6)	
F2	40 (12.0)	24 (21.6)	
F3	56 (16.8)	20 (18.0)	
F4	133 (40.1)	43 (38.8)	

Except where indicated, data are numbers of patients, with percentages in parentheses

*ALT* alanine transferase, *APRI* aspartate transaminase-to-platelet ratio, *AST* aspartate transaminase, *FIB-4* fibrosis-4 index, *HCC* hepatocellular carcinoma, *INR* international normalized ratio, *NAFLD* non-alcoholic fatty liver diseases

*Data are medians, with interquartile range in parentheses

### Reference standard for liver fibrosis

Liver pathologic examination served as the reference standard for staging liver fibrosis. Liver specimens were obtained by liver resection (*n* = 308); liver transplantation (*n* = 32); or percutaneous liver biopsy (*n* = 103) (Table 1), which were histologically analyzed by two pathologists in consensus. Fibrosis stage was determined according to the Metavir scoring system [14], as follows: F0, no fibrosis; F1, portal fibrosis without septa; F2, portal fibrosis with rare septa; F3, numerous septa without cirrhosis; F4, cirrhosis. F ≥ 2 was considered as significant fibrosis and F ≥ 3 as advanced fibrosis.

### Serum fibrosis tests

The aspartate aminotransferase-to-platelet ratio index (APRI) and the fibrosis-4 index (FIB-4) were calculated as (aspartate aminotransferase [international units/liter]/upper normal limit × 100/platelet counts [× 10^9^/liter]) and (age [years] × aspartate aminotransferase [international units/liter])/(platelet counts [× 10^9^/liter] × alanine aminotransferase [international units/liter]^1/2^) [15, 16], respectively. These indices were calculated using the results of laboratory tests performed within 26 days ± 13 (range 3–76 days) from obtaining results of pathologic examination of the liver.

### CT image acquisition

Contrast-enhanced CT scans were acquired in the axial plane with 0.75–1.5-mm-thick sections and a 0.75–1.5-mm reconstruction interval. Image acquisition parameters are detailed in Appendix E1 (online resource).

### Radiomic feature extraction and selection

**Fig. 2:**
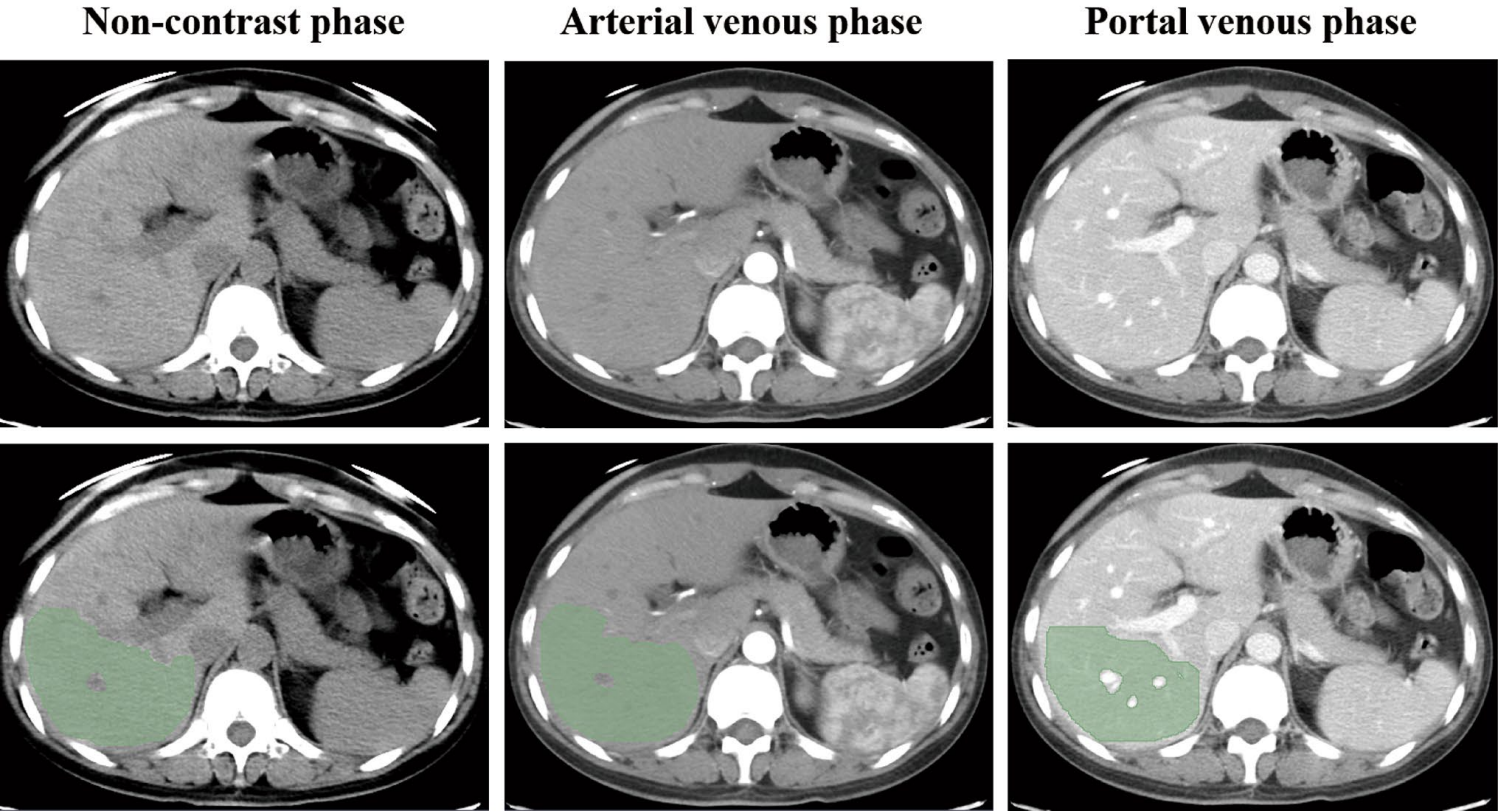
ROIs for the liver at contrast-enhanced CT. ROIs were delineated along the margin of the right hepatic lobe, at the level of the right portal vein, by excluding large hepatic vessels and masses on non-contrast, arterial and portal venous phases CT images. *CT* computed tomography, *ROI* region of interest

One reader (S.N.T., with 7 years of clinical experience in abdominal radiology) selected regions of interest (ROIs) in the liver of all patients. ROIs for the liver were delineated along the margin of the right hepatic lobe, at the level of the right portal vein, by excluding large hepatic vessels and masses on non-contrast (mean area of ROIs, 48 cm^2^ ± 16; range 17–108 cm^2^), arterial (mean area of ROIs, 48 cm^2^ ± 18; range 16–108 cm^2^) and portal (mean area of ROIs, 51 cm^2^ ± 18; range 16–109 cm^2^) venous phases CT images using 3D slicer (version 4.11.1; http://www.slicer.org) (Fig. 2). To explore the stability of each feature, 30 patients were randomly chosen; reader 1 repeated image segmentation twice and reader 2 independently performed segmentation to evaluate the intra- and interobserver reproducibility. The reproducibility was quantified by the intraclass correlation coefficient (ICC).

Image preprocessing and feature extraction were performed using the open-source Pyradiomics package (version 2.2.0: http://www.radiomics.io/pyradiomics.html). The voxel spacing was standardized with the size of 1 × 1 × 1 mm and voxel intensity values were discretized with a bin width of 25 HU to reduce the interference of image noise and normalize intensities [17], respectively. We extracted 837 radiomic features (18 first-order statistics, 75 textural features and 744 wavelet transformations) from each two-dimensional segmentation, giving a total of 2511 for every phase CT images (non-contrast, arterial and portal venous phases). The *z*-scores were used to standardize values of features and the mean and standard deviation determined in the training cohort were applied in the validation cohort.

A three-step procedure was followed to select significant radiomic features. First, the reliability of each feature was qualified using ICC and features with ICC more than 0.9 were kept for further analysis [18]. Second, irrelevant features that weakly correlated with fibrosis stage were removed; the correlation between each radiomic feature and metavir fibrosis stage was evaluated using the Kendall correlation coefficient. Features with correlation coefficients less than 0.15 were eliminated. The final step in feature selection was performed using the least absolute shrinkage and selection operator (LASSO) logistic regression algorithm with penalty parameter tuning conducted by tenfold cross-validation [19], between stages F0–F2 and F3–F4, and features with nonzero coefficients were considered independently related to fibrosis stage.

### Clinical factors selection

We devised a three-step procedure for selection of clinical factors. First, we used kendall correlation analysis to screen out factors with significant correlation (kendall correlation analysis, *p* < 0.05). Second, forward conditional logistic multivariable analysis was used to select factors for the discrimination between stages F0–F2 and F3–F4 (input and output *p* value: 0.05 and 0.1, respectively). Third, a function on the basis of the variance inflation factor (VIF) was conducted to check for the collinearity of variables included in the regression equations [20]. Variables with VIF greater than 10 (indicating multicollinearity) were excluded.

### Model establishment and validation

The radiomics signature for the prediction of fibrosis (R-score) was created using support vector machine (SVM) as a multi classification to distinguish among stages F0, F1, F2, F3 and F4. SVM can be used to carry out general regression and classification and it was performed using “e1071” package (https://CRAN.R-project.org/package=e1071) on R software (version 3.6.1, http://www.r-project.org).

Multivariate linear regression analysis (Appendix E2 in online resource) was performed to establish a final model (R-fibrosis) based on radiomics signature (R-score) and clinical factors for the prediction of fibrosis. The performance of models was tested in the independent validation cohort using the equation derived from the training cohort.

### Statistical analysis

Categorical and continuous variables were compared using χ^2^ test and the Mann–Whitney *U* test, respectively. The correlation between results calculated from models and pathologic liver fibrosis stage was evaluated using the spearman correlation analysis. Performance of models for staging liver fibrosis was evaluated using receiver operating characteristics (ROC) curve analysis, area under the curve (AUC) value and the Obuchowski index, a multinomial version of ROC curve analysis adapted for ordinal references such as metavir staging of liver fibrosis [21]. The Obuchowski index is a weighted average of the areas under the curve obtained for all possible pairs of fibrosis stages to be differentiated and it estimates the probability that a test will correctly rank two randomly chosen patients with different stages of fibrosis. The optimal thresholds of models were determined using the ROC analysis by maximizing the Youden index. Delong nonparameteric approach was used to compare AUC values [22]. Calibration curves were plotted to evaluate the calibration of the established model, accompanied by the Hosmer–Lemeshow test. Additionally, a decision curve analysis (DCA) was performed to assess the clinical usefulness and net benefits of the developed radiomics models [23]. A two-sided *p* value less than 0.05 was indicative of a statistically significant difference.

## Results

### Characteristics of the study cohorts

The baseline characteristics of all patients are summarized in Table 1. There were no significant differences in clinical and pathological characteristics between the training and validation cohorts. No differences were found in rates of significant fibrosis (Training: 69.0%, 229 of 332; Validation: 78.4%, 87 of 111; *p* = 0.06), serious fibrosis (Training: 56.9%, 189 of 332; Validation: 56.8%, 63 of 111; *p* = 0.97) and cirrhosis (Training: 40.1%, 133 of 332; Validation: 38.8%, 43 of 111; *p* = 0.81) between the two cohorts. Results of APRI and FIB-4 were similar (*p* > 0.05 for both) between the two study cohorts.

### Fibrosis-related clinical factors

In the training cohort, platelet (PLT) count, glutamyl transpeptidase (GGT), albumin (ALB), albumin to globulin ratio (A/G) and total cholesterol (TC) were identified as independent fibrosis predictors by the multivariable logistic regression analysis (Table 2). The VIF of TC was 10.7 (over 10), indicating the collinearity, in which the variable should be excluded. According to the Kendall correlation coefficient of GGT and PLT (0.15 and − 0.292, respectively), the GGT to PLT ratio was involved in the prediction model.

**Table 2:** Clinical characteristics of the training cohort related to fibrosis

Variables	Kendall correlation analysis		Multivariable analysis		Collinearity Statistics	
	Coefficient	*p* value	*b* coefficient	*p* value	Tolerance	VIF
Age (years)	− 0.042	0.33	NA	NA	NA	NA
Sex (male, female)	0.087	0.10	NA	NA	NA	NA
RBC (10^9^/L)	− 0.107	0.01	NA	0.53	0.284	3.52
PLT (10^9^/L)	− 0.292	< 0.001	− 0.012	< 0.001	0.699	1.43
Hb (g/L)	− 0.106	0.02	NA	0.66	0.276	3.63
ALT (U/L)	0.089	0.04	NA	0.54	0.123	8.10
AST (U/L)	0.164	< 0.001	NA	0.98	0.115	8.68
ALP (U/L)	0.147	0.001	NA	0.81	0.202	4.95
GGT (U/L)	0.150	0.001	0.007	0.004	0.469	2.13
LDH (U/L)	0.045	0.298	NA	NA	NA	NA
TB (umol/L)	0.000	0.99	NA	NA	NA	NA
CB (umol/L)	0.169	< 0.001	NA	0.23	0.104	9.59
ALB (g/L)	− 0.117	0.007	0.139	0.002	0.491	2.04
GLOB (g/L)	0.081	0.06	NA	NA	NA	NA
A/G	− 0.104	0.02	− 1.668	< 0.001	0.693	1.44
TBA (umol/L)	0.213	< 0.001	NA	0.22	0.528	1.89
LAP (U/L)	0.056	0.20	NA	NA	NA	NA
TC (mmol/L)	− 0.184	< 0.001	− 0.399	0.02	0.093	10.70
HDL-C (mmol/L)	0.009	0.83	NA	NA	NA	NA
LDL-C (mmol/L)	− 0.171	< 0.001	NA	0.20	0.045	22.03
Apo A1 (g/L)	0.010	0.83	NA	NA	NA	NA
Apo B (g/L)	− 0.168	< 0.001	NA	0.63	0.087	11.48
CRP (mg/L)	0.101	0.02	NA	0.49	0.512	1.95
PT (s)	0.189	< 0.001	NA	0.40	0.017	59.27
INR	0.210	< 0.001	NA	0.35	0.017	60.28

*b* coefficients are from multivariable logistic regression. Clinical variables found to be significantly related to cirrhosis through spearman correlation analysis entered into forward conditional logistic multivariate analysis

*ALB* albumin, *ALP* alkaline phosphatase, *ALT* alanine aminotransferase, *Apo A1* apolipoprotein A1, *Apo B* apolipoprotein B, *AST* aspartate aminotransferase, *A/G* albumin to globulin ratio, *CB* conjugated bilirubin, *CRP* C reactive protein, *GGT* glutamyl transpeptidase, *GLOB* globulin, *Hb* hemoglobin, *HDL-C* high density lipoprotein cholesterol, *INR* international normalized ratio, *LAP* leucine arylamidase, *LDH* lactate dehydrogenase, *LDL-C* low density lipoprotein cholesterol, *PLT* blood platelet, *PT* prothrombin time, *RBC* red blood cell, *TB* serum total bilirubin, *TBA* total bile acid, *TC* total cholesterol, *VIF* variance inflation factor

### Radiomic feature selection and signature construction

**Fig. 3:**
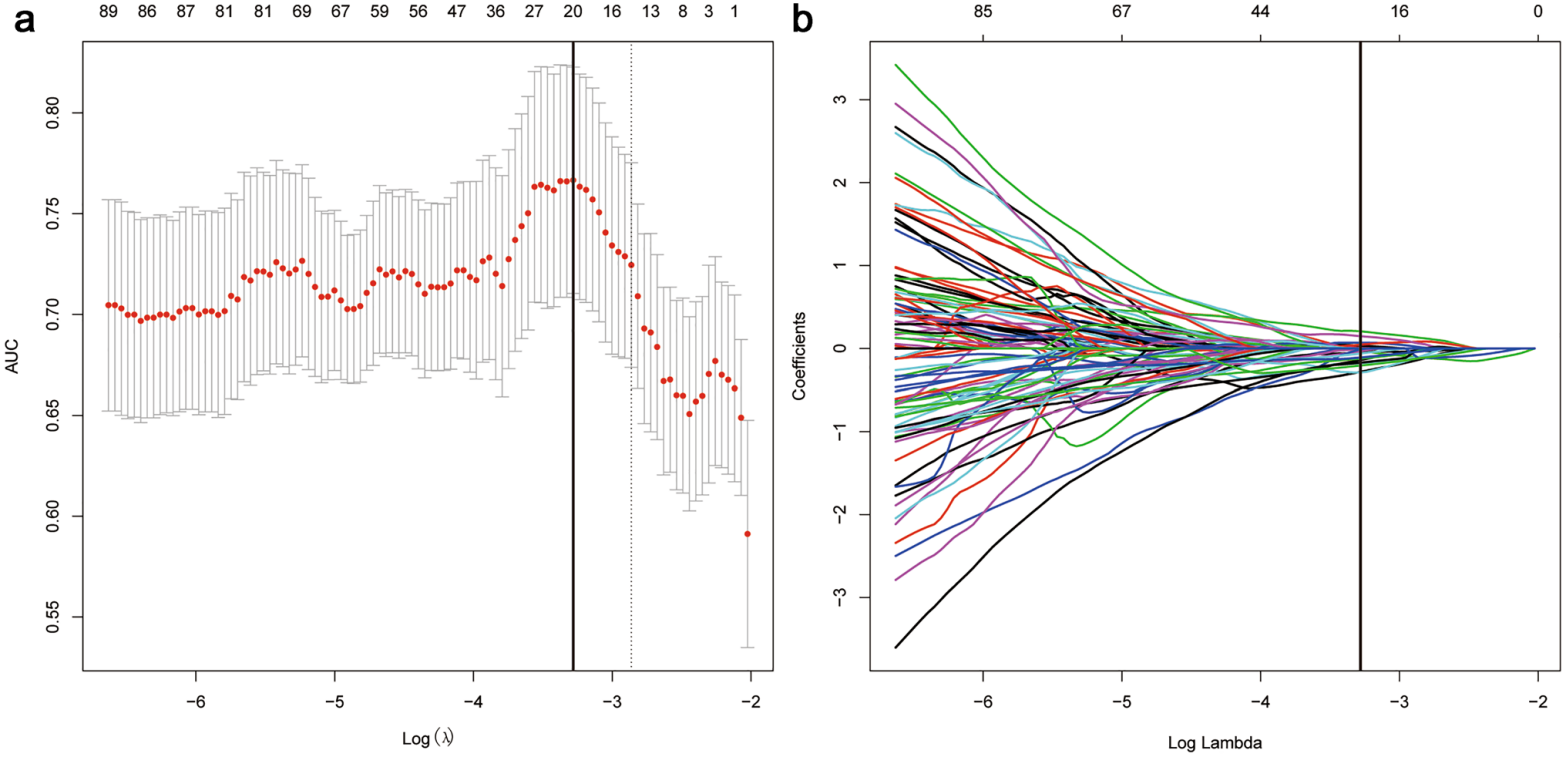
Selections of radiomic features using the LASSO regression. **a** Optimal λ value was determined by the LASSO model using tenfold cross-validation via minimum criteria. The AUC curve was plotted versus log(λ). Dotted vertical lines were drawn at the optimal values using the minimum criteria and the 1 standard error of the minimum criteria (the 1—standard error criteria). The optimal λ value of 0.0376 was chosen. **b** LASSO coefficient profiles of the 320 selected features is presented. *AUC* area under the curve. *LASSO* least absolute shrinkage and selection operator

Among 2084 radiomic features with high stability, 320 features with significant correlations to fibrosis stage were identified. And then, 21 independent features with nonzero coefficients were finally selected by the LASSO logistic regression (Fig. 3). A radiomic signature was constructed using SVM algorithm. The type of SVM was “eps-classification”, of which the kernel function was radial basis. The value of gamma and epsilon was 0.045 and 0.1, respectively. The total number of support vectors was 153.

**Fig. 4:**
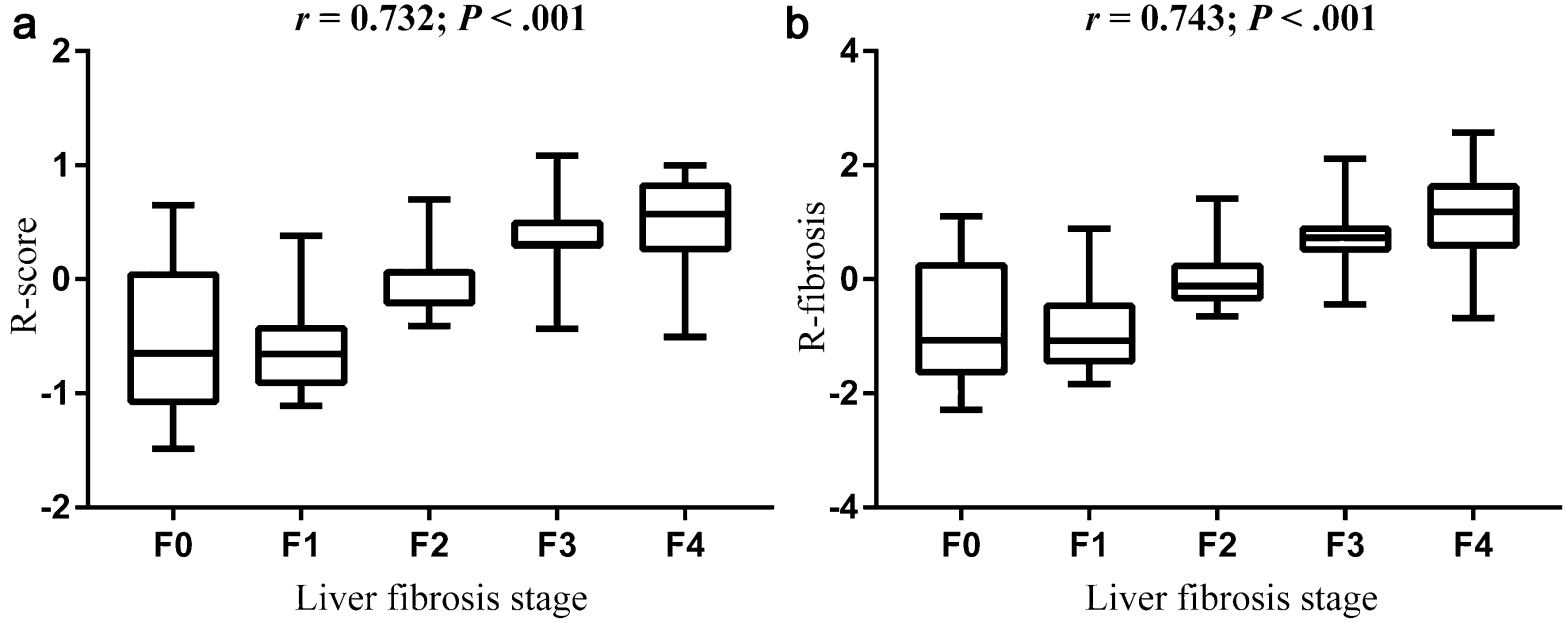
Box-and-whisker plot of the *R*-score and R-fibrosis for each pathologic liver fibrosis stage in the training cohort. Boxes, thick horizontal bars within the boxes, and whiskers represent interquartile ranges (IQRs), medians and 1.5 × IQR, respectively. Both *R*-score and R-fibrosis have positive correlations with liver fibrosis stage (*r* > 0.7, *p* < 0.001 for both)

The R-score showed a positive correlation with liver fibrosis stage (*r* = 0.717, *p* < 0.001; Fig. 4). In the training cohort, the R-score showed AUCs of 0.904 (95% confidence interval [CI] 0.865, 0.942), 0.911 (95% CI 0.880, 0.943) and 0.844 (95% CI 0.800, 0.889) for the diagnosis of significant fibrosis, advanced fibrosis and cirrhosis. The Obuchowski index, which indicates the overall accuracy of multiclass liver fibrosis staging, was 0. 847 (95% CI 0.797, 0.897) (Table 3). The R-score showed favorable discriminatory ability, with AUCs of 0.875 (95% CI 0.781, 0.969), 0.900 (95% CI 0.842, 0.959) and 0.857 (95% CI 0.790, 0.925), for significant fibrosis, advanced fibrosis and cirrhosis in the validation cohort (Table 4).

**Table 3:** Diagnostic performance of models for staging liver fibrosis in the training cohort

Parameter	*R*-score	R-fibrosis
Significant fibrosis (F2–F4)		
AUC	0.904 (0.865, 0.942)	0.905 (0.867, 0.943)
Threshold	− 0.258	− 0.403
Sensitivity (%)	92.1 (87.9, 95.3)	94.8 (91.0, 97.3)
Specificity (%)	76.7 (67.3, 84.5)	74.8 (65.2, 82.8)
Accuracy (%)	87.3 (83.9, 90.9)	88.6 (85.1, 92.0)
Advanced fibrosis (F3–F4)		
AUC	0.911 (0.880, 0.943)	0.915 (0.884, 0.946)
Threshold	0.167	0.332
Sensitivity (%)	83.6 (77.4, 88.7)	85.8 (79.9, 90.5)
Specificity (%)	89.3 (83.1, 93.7)	87.9 (81.6, 92.7)
Accuracy (%)	86.1 (82.4, 89.9)	86.7 (83.1, 90.4)
Cirrhosis (F4)		
AUC	0.844 (0.800, 0.889)	0.857 (0.814, 0.899)
Threshold	0.503	0.950
Sensitivity (%)	60.7 (50.8, 70.0)	65.4 (55.6, 74.4)
Specificity (%)	95.6 (92.0, 97.8)	90.2 (85.6, 93.8)
Accuracy (%)	84.3 (80.4, 88.3)	82.2 (78.1, 86.4)
Obuchowski index	0.847 (0.797, 0.897)	0.852 (0.807, 0.898)

Data in parenthesis are 95% confidence intervals

*AUC* area under the curve, *R-score* radiomics signature for the prediction of fibrosis, *R-fibrosis* final established model for the prediction of fibrosis

**Table 4:** Areas under the curve and Obuchowski indexes of *R*-score and serum fibrosis tests for staging liver fibrosis in the validation cohort

Parameter	*R*-score	R-fibrosis	APRI	FIB-4
Significant fibrosis (F2–F4)	0.875 (0.781, 0.969)	0.901 (0.818, 0.984)	0.692 (0.581, 0.804) ^*§^	0.713 (0.619, 0.808) ^*§^
Advanced fibrosis (F3–F4)	0.900 (0.842, 0.959)	0.883 (0.822, 0.945)	0.673 (0.569, 0.776) ^*§^	0.714 (0.617, 0.811) ^*§^
Cirrhosis (F4)	0.857 (0.790, 0.925)	0.860 (0.791, 0.930)	0.653 (0.533, 0.772) ^*§^	0.701 (0.581, 0.820) ^*§^
Obuchowski index	0.843 (0.808, 0.877)	0.846 (0.812, 0.880)	0.651 (0.561, 0.742) ^*§^	0.676 (0.606, 0.780) ^*§^

Data in parenthesis are 95% confidence intervals

*APRI* aspartate aminotransferase-to-platelet ratio index, *AUC* area under the curve, *FIB-4* fibrosis-4 index, *R-score* radiomics signature for the prediction of fibrosis, *R-fibrosis* final established model for the prediction of fibrosis

*Significantly different from the results of R-score (*p* < 0.05)

§Significantly different from the results of R-fibrosis (*p* < 0.05)

### Development and validation of the prediction model

A prediction model (R-fibrosis) integrated the R-score, GGT to PLT ratio, A/G and ALB (0, > 40 g/L; 1, ≤ 40 g/L). The equation for R-fibrosis derived from the training cohort was:

R-fibrosis = 1.675 × R-score + 0.107 × GGT/PLT + 0.121 × ALB × A/G.

The R-fibrosis showed a positive correlation with liver fibrosis stage (*r* = 0.743, *p* < 0.001; Fig. 4). In the training cohort, AUCs of R-fibrosis for diagnosing significant fibrosis, advanced fibrosis and cirrhosis were 0.905 (95% CI 0.867, 0.943), 0.915 (95% CI 0.884, 0.946) and 0.915 (95% CI 0.884, 0.946), respectively. And the Obuchowski index was 0.852 (95% CI 0.807, 0.898).

**Fig. 5:**
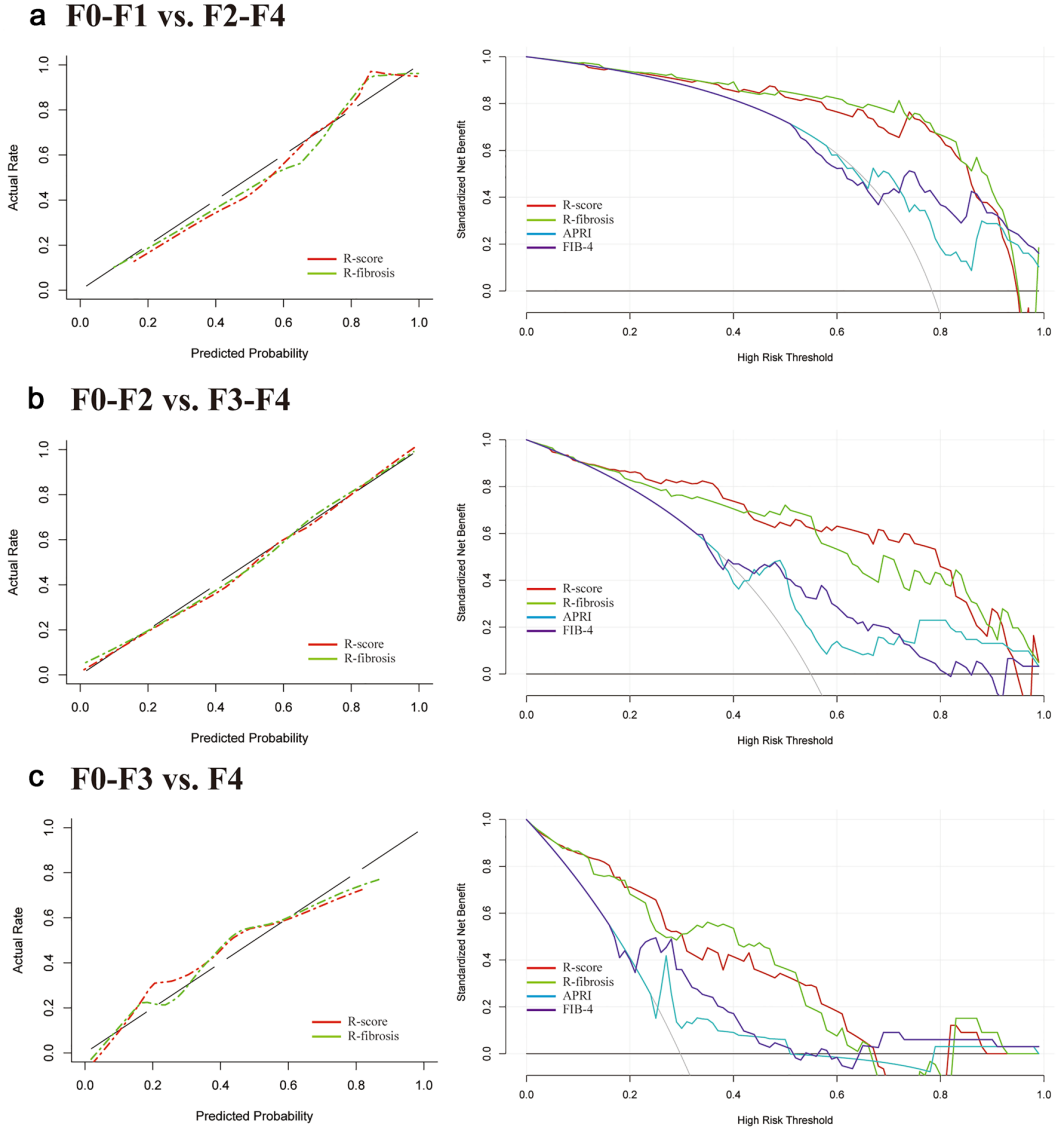
Calibration curves (left) and decision curve analysis (right) for each model in the validation dataset. *R*-score and R-fibrosis were established due to the training cohort and validated for the prediction of significant fibrosis (**a**), advanced fibrosis (**b**) and cirrhosis (**c**). In decision curve analysis, the *y*-axis measures the net benefit, which was calculated by summing the benefits (true-positive results) and subtracting the harms (false-positive results), weighting the latter by a factor related to the relative harm of an undetected fibrosis status compared with the harm of unnecessary treatment

Table 4 summarizes the diagnostic performance of R-fibrosis, R-score, APRI and FIB-4 in the validation cohort. The AUCs of R-fibrosis and R-score for aiding in diagnosis of significant fibrosis (0.901 and 0.875, 95% CI [0.818, 0.984] and [0.781, 0.969]), advanced fibrosis (0.883 and 0.900, 95% CI [0.822, 0.945] and [0.842, 0.959]) and cirrhosis (0.860 and 0.857, 95% CI [0.791, 0.930] and [0.790, 0.925]) were higher than the AUCs for APRI (range 0.653–0.692) and FIB-4 (range 0.701–0.714) (Fig. 5). Using thresholds determined in the training cohort, R-fibrosis and R-score had a sensitivity range of 70–87%, specificity range of 71–97%, and accuracy range of 82–86% in diagnosing significant fibrosis, advanced fibrosis and cirrhosis in the validation cohort (Table 5).

**Table 5:** Diagnostic performance of models for staging liver fibrosis in the validation cohort

Parameter	Model	Threshold value*	Sensitivity (%)	Specificity (%)	Accuracy (%)
Significant fibrosis (F2–F4)	R-score	− 0.258	83.9 (73/87) [76.0, 91.8]	87.5 (21/24) [73.2, 99.9]	84.7 (94/111) [77.9, 91.5]
	R-fibrosis	− 0.403	87.4 (76/87) [80.2, 94.5]	70.8 (17/24) [51.2, 90.4]	83.8 (93/111) [76.8, 90.7]
Advanced fibrosis (F3–F4)	R-score	0.167	71.4 (45/63) [60.0, 82.9]	95.8 (46/48) [90.0, 99.9]	82.0 (91/111) [74.7, 89.2]
	R-fibrosis	0.332	76.2 (48/63) [65.4, 87.0]	91.7 (44/48) [83.6, 99.8]	82.9 (92/111) [75.8, 90.0]
Cirrhosis (F4)	R-score	0.503	69.8 (30/43) [55.5, 84.1]	91.2 (62/68) [84.3, 98.1]	82.9 (92/111) [75.8, 90.0]
	R-fibrosis	0.950	79.1 (34/43) [66.4, 91.7]	89.7 (61/68) [82.3, 97.1]	85.6 (95/111) [78.9, 92.2]

Data in parenthesis are numerator/denominator and data in brackets are 95% confidence interval

*Threshold values were derived from the training cohort

Calibration curves of the R-fibrosis and R-score demonstrated great agreement between predicted and actual significant fibrosis, advanced fibrosis and cirrhosis in the validation cohort (Fig. 5). The Hosmer–Lemeshow test yielded a *p* value of > 0.05, suggesting no departure from the good fit. The decision curve analysis for the R-fibrosis, R-score, APRI and FIB-4 are presented in Fig. 5. R-fibrosis and R-score provided higher net benefit compared with other models and simple strategies of all patients or no patients across the majority of the range of reasonable threshold probabilities in the validation cohort. No obvious differences were found in terms of clinical benefit between R-fibrosis and *R*-score.

## Discussion

The aim of this study was to develop and validate radiomics-based models on contrast-enhanced CT radiomics for liver fibrosis. We concluded that radiomics analysis of contrast-enhanced CT allows for more accurate staging of liver fibrosis compared with other models. The R-fibrosis and R-score created by the training cohort data predicted the staging of liver fibrosis in the validation cohort with AUCs of 0.84–0.90 and accuracies of 82–86%. In agreement with our hypothesis, radiomics models (Obuchowski index, 0.84–0.85) outperformed custom serum indices (Obuchowski index, 0.65–0.68).

There are various less-invasive methods for staging liver fibrosis including serological markers and elastography. Ultrasound-based elastography (including transient elastography [TE] and two-dimensional shear wave elastography [2D-SWE]) and magnetic resonance elastography (MRE) are known to have great diagnostic performance for staging liver fibrosis [19, 24, 25]. However, elastography techniques are not widely used in China because of high prices and limited cost-effectiveness for general hospitals. HBV carriers are frequently suggested to receive annual contrast-enhanced CT or MRI in China. Our previous study developed a radiomics-based model at non-contrast CT for predicting cirrhosis [13] and this study used contrast-enhanced CT for further investigation (significant & advanced fibrosis). Ultrasonography is used as the initial tool for early screening of liver tumor in patients with chronic hepatitis in the world. However, the normalization of ultrasound images is difficult and software that can preprocess and extract radiomic features from two-dimensional images is rare. We are also researching the image processing algorithms for future consideration of ultrasound images.

This study considered not only chronic liver diseases but also liver masses to make the R-fibrosis and *R*-score suitable for major kinds of patients with liver fibrosis. Fibrosis staging can help guide the treatment plans. Both contrast-enhanced CT and MRI are recommended by guidelines for early detection of liver tumor for patients with chronic liver diseases [7, 26], and many studies have focused on image data mining at MRI involving image findings and texture analysis [27–29]. A study conducted by Park et al. [29] analyzed Gadoxetic Acid-enhanced MRI for staging liver fibrosis using radiomics and obtained radiomics fibrosis index with the AUC range of 0.89–0.91 (similar to *R*-score and R-fibrosis). Actually, CT is more readily available than MRI. Computer-aided visual assessment of liver or spleen volume and homogeneity on CT allowed for the detection of fibrosis stage but showed neglect of multiclass accuracy [10, 30]. Moreover, none of them were validated in independent test data sets. A deep convolutional neural network (DCNN) system for staging liver fibrosis was developed using portal venous phases CT images [31]. Unlike texture analysis, the DCNN system extracted and analyzed features from cropped and zoomed images. Diagnostic performance of the DCNN system is not greater than us (AUC range 0.73–0.76), although there should be a head-to-head comparison for comparing these two methods. A recent study revealed that DCNN system should be established based on the entire upper abdomen at CT images which can significantly improve diagnostic performance (AUC range 0.88–0.92) [32].

The established radiomic signature (*R*-score) in this study included 4 first-order statistics and 17 textural features. As similar to previous studies [33, 34], most of (90.5%, 19 of 21) these included features were processed by wavelet transform. 12 features were derived from non-contrast CT and others were from arterial (4) and portal (5) venous phases, of which the cause might be non-contrast CT can provide more stable features without effects of personal intake. The final model (R-fibrosis) included GGT/PLT, ALB and A/G in addition to *R*-score. Results calculated by R-score are decision values of all binary classifiers computed in multiclass classification. It is normal for established models to get negative values. We aimed to develop models with detailed cutoff values for multiclass classification in this study to be easily applied in other centers. The predictive value of the GGT to PLT ratio for significant fibrosis and cirrhosis was confirmed by Lemoine et al. [35] and Lu et al. [36]. ALB has been confirmed as an independent indicator of advanced liver fibrosis in patients with NAFLD [37], and it can also significantly contribute to the index for staging liver fibrosis in patients with viral hepatitis [38, 39]. A/G was used as biomarkers in many cases such as tumor prognosis [40–42] and chronic diseases [43–45], but only one study involved A/G into the fibrosis markers [46]. The specificity of A/G for fibrosis might not be so high, and thus we make it computable when ALB ≤ 40 g/L.

There were several limitations in our study. First, the limited population size and the unbalanced distribution of the patient population restricted the great establishment of the prediction model. Moreover, the retrospective study may introduce selection biases, and there were larger numbers of patients with advanced fibrosis (i.e. stages F3&F4) than others (i.e. stages F0–F2). Second, the proposed radiomics-based model was established using data obtained from a single center. Our model needed to be further validated by prospective multicenter studies with considerably large datasets. Third, image findings related to significant fibrosis (a nodular or irregular hepatic surface, parenchymal abnormalities, a blunt liver edge, intrahepatic morphological changes and clinical manifestations of portal hypertension) were not considered in this study. The main reason is that these image findings are frequently suggestive of cirrhosis [47]. Fourth, this study did not consider different etiologies on feature extraction. Different etiologies have a certain impact on fibrosis, indicating the possibility of different feature values caused by different etiologies. Therefore, subgroup analysis should be conducted in different etiologies to ensure the objectivity of the results. Finally, because elastography methods (TE or 2DSWE) were not performed for these patients, we were unable to compare the efficacy of our model with that of elastography for staging liver fibrosis.

## Conclusions

In conclusion, we proposed a noninvasive and convenient radiomics-based model at contrast-enhanced CT images which allowed for accurate diagnosis of clinically significant liver fibrosis. Compared with our previous radiomics model based on non-contrast CT scans, R-fibrosis can additionally become as an update version for the prediction of significant and advanced fibrosis.

## Supplementary Information

Below is the link to the electronic supplementary material.

Supplementary file1 (DOCX 17 KB)

## Data Availability

The data are not available because of patients’ privacy.

## Abbreviations

CT: Computed tomography
NAFLD: Non-alcoholic fatty liver diseases
AASLD: American Association for the Study of Liver Diseases
HBV: Hepatitis B virus
APRI: Aspartate aminotransferase-to-platelet ratio index
FIB-4: Fibrosis-4 index
ROI: Region of interest
ICC: Intraclass correlation coefficient
LASSO: Least absolute shrinkage and selection operator
VIF: Variance inflation factor
R-score: Radiomics signature for the prediction of fibrosis
SVM: Support vector machine
R-fibrosis: Final model based on radiomics signature and clinical factors for the prediction of fibrosis
ROC: Receiver operating characteristics
AUC: Area under the curve
DCA: Decision curve analysis
PLT: Platelet
GGT: Glutamyl transpeptidase
ALB: Albumin
A/G: Albumin to globulin ratio
TC: Total cholesterol
CI: Confidence interval
TE: Transient elastography
2D-SWE: Two-dimensional shear wave elastography
MRE: Magnetic resonance elastography
DCNN: Deep convolutional neural network
