# Supplementary material for: Radiomics analysis of contrast-enhanced CT for staging liver fibrosis: an update for image biomarker
Source: Hepatol Int. 2022 Mar 28;16(3):627–39. doi: 10.1007/s12072-022-10326-7 (PMC9174317; doi:10.1007/s12072-022-10326-7)
Supplement: Supplementary file 1 — Supplementary file1 (DOCX 17 KB) [file 12072_2022_10326_MOESM1_ESM.docx]

**Appendix E1**

**Technical Details of CT Image Acquisition**

Multiphasic contrast-enhanced CT scan with the multidetector spiral CT (Lightspeed, VCT, or Discovery HD 750, GE Healthcare, US) were performed on all patients, and the scanning range covered the upper abdomen or the entire abdomen. After non-contrast enhanced CT scan, a 2 mL/kg bodyweight contrast media (Omnipaque 350 mgI/mL; GE Healthcare) injected through the antecubital vein (4 mL/s) with a power injector (Medrad tellant, Indianola, PA, US), followed by 40 mL of saline solution (4 mL/s). At 30 and 60s, the arterial phase and portal venous phase images were obtained. The CT scan parameters: the tube voltage (120 kVp), tube current (250–350 mA), collimating slice thickness (5 mm), reconstruction slice thickness (1.25 mm), slice interval (5 mm), rotation time (0.6 s), helical pitch (1.375), field of view (35–40 cm), and matrix (512 × 512). A standard reconstruction algorithm was applied.

**Appendix E2**

**Details of** **multi****variate linear regression analysis**

Multiple linear regression analysis is an extension of simple linear regression analysis, which can be used to assess the association between two or more independent variables and a single dependent variable. The multiple linear regression equation is as follows:


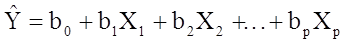


Independent variables in regression models can be continuous or dichotomous. Regression models can also accommodate categorical independent variables.
